# Novel perceptions of the involvement of CPZ in gastric cancer prognosis and immunomodulation

**DOI:** 10.3389/fonc.2025.1599542

**Published:** 2025-09-10

**Authors:** ZhenMin Yuan, XiaoYing Yang, JunJie Huang, JunRen Wei, Lei Tian

**Affiliations:** ^1^ Department of Gastrointestinal & Gland Surgery Division I, The First Affiliated Hospital of Guangxi Medical University, Nanning, Guangxi, China; ^2^ Hepatobiliary and Endocrine Surgery Department, Beihai People’s Hospital, Beihai, China; ^3^ Breast Surgery Department, Qinzhou First People’s Hospital, Qinzhou, China; ^4^ The First Clinical Medical College, Guangxi Medical University, Nanning, Guangxi, China

**Keywords:** gastric cancer, CPZ, bioinformatics analysis, immune regulation, prognosis

## Abstract

**Background:**

Gastric cancer (GC) is a highly malignant tumor with a complex etiology. Most patients are diagnosed at an advanced stage with poor prognosis. The carboxypeptidase family is associated with progression in many cancers. Carboxypeptidase Z (CPZ) is a cellular matrix regulator. Corresponding studies on CPZ expression and the molecular mechanisms of GC prognosis and immunomodulation are lacking. We examined the influence of CPZ expression on the prognosis and immunomodulation of GC and the corresponding clinical significance.

**Methods:**

CPZ gene expression in pan-cancer analysis was conducted using the Tumor Immune Estimation Resource (TIMER2.0) database. Differences in CPZ expression levels were investigated using 412 GC samples and 36 normal tissue samples from The Cancer Genome Atlas (TCGA) database. These results were validated using the Gene Expression Profiling Interactive Analysis (GEPIA2) and Gene Expression Omnibus (GEO) datasets GSE65801 and GSE103236. The prognostic and diagnostic value of CPZ expression in patients with GC was assessed using Kaplan-Meier plotter, the chi-square test, and the receiver operating characteristic (ROC). Genes with joint CPZ differential expression were identified for functional enrichment analysis according to TCGA-STAD database. The link between CPZ and immune cell infiltration, immune checkpoints, and fibroblasts was determined using CIBERSORT, single-sample gene set enrichment analysis, and the TIMER2.0 immuno-gene module. The tumor mutational burden and immunotherapy were analyzed using maftools and The Cancer Imaging Archive data. CPZ expression-related drug susceptibility was analyzed using R oncoPredict package and Wilcoxon tests. Differential CPZ expression in cancer and paracancerous tissues was verified using immunohistochemistry (IHC) and quantitative PCR (qPCR).

**Results:**

The analysis demonstrated significantly increased CPZ expression in GC tissues. The CPZ expression level was an independent GC prognostic factor of risk. CPZ expression influenced immune cell and fibroblast infiltration in the GC tumor microenvironment. Elevated CPZ expression led to patient resistance to common chemotherapeutic agents such as oxaliplatin, docetaxel, and cisplatin. IHC and qPCR demonstrated significantly increased CPZ expression in GC tissues.

**Conclusion:**

Elevated CPZ expression in GC tissues affects patient survival prognosis and can increase immune cell infiltration, affecting the tumor microenvironment. CPZ may be a novel predictive biomarker associated with immune-modulated prognosis in GC.

## 1 Introduction

Stomach adenocarcinoma (STAD) is a malignant tumor that seriously threatens human life, the fifth most prevalent malignancy globally, and the fourth most frequent reason for death from cancer worldwide (1). Gastric cancer (GC) treatment primarily involves surgical resection with curative intent, supplemented by perioperative chemotherapy, hyperthermic intraperitoneal chemotherapy, molecularly targeted therapies, and immunotherapy. Given the insidious onset of GC in its early stages and low rates of regular screening, most patients receive their diagnosis at an advanced disease stage (2). Advanced GC is highly malignant, grows rapidly, and metastasizes easily. Some patients with advanced GC are deprived of the opportunity for surgery and can only improve their survival chances through neoadjuvant chemotherapy, palliative chemotherapy, or immunotherapy (3). As GC is heterogeneous, its gene expression, tumor immune microenvironment (TIME), and tumor cell composition feature complex differences (4). GC carcinogenesis is a disease process involving multigene molecules, and its progression is closely related to abnormal gene expression (5). Furthermore, the availability of potentially relevant predictive biomarkers (HER2, VEGF, FGFR2) and immune checkpoints (T cell immunoreceptor with Ig and ITIM domains [TIGIT], programmed cell death 1 [PD1], cytotoxic T-lymphocyte-associated protein 4 [CTLA4]) (6) in GC provides limited assistance in its diagnosis and treatment. Collectively, these factors contribute to the substantial global burden of GC. Therefore, research identifying more accurate prognostic and predictive genetic markers for GC is urgently needed.

The metallocarboxypeptidase gene family comprises extracellular enzymes that promote protein digestion and are classified into carboxypeptidase A and B (CPA/B) and carboxypeptidase N and E (CPN/E) subgroups based on amino acid sequence differences, functions, and mechanisms of action. The members of the CPA/B subfamily are included in digesting food and degrading various proteins. Contrastingly, CPN/E subfamily members exhibit distinct structural and pH activity differences (7–10), conferring higher selectivity and a role in modulating cellular messengers (11). Carboxypeptidase Z (CPZ) belongs to the CPN/E subgroup and is located on human chromosome 4p16.1 (https://pmc.ncbi.nlm.nih.gov/) (12, 13). CPZ is a secreted enzyme with optimal activity when the pH is neutral (pH 7.5), exhibiting high affinity for binding to the extracellular matrix (ECM) (14). CPZ functions by cleaving C-terminal basic amino acids, and specifically cleaves C-terminal arginine (Arg) residues (12). The family of carboxypeptidases is closely linked to progression in various cancers. CPE is implicated in pancreatic cancer, lung adenocarcinoma, and osteosarcoma progression, and downregulating it inhibits the proliferation and metastasis of these tumors (15). Knocking down CPN suppresses breast cancer cell invasion and migration (16). CPZ is significantly associated with neuroblastoma risk (13). The CPZ gene produces significant immunohistochemical (IHC) staining in the tissues of adrenocorticotropic hormone-synthesizing suprarenal cortical cell adenomas, adenomas of growth hormone (GH)-producing GH cells, and adenomas of prolactin-producing lactation cells (17). CPE may promote epithelial–mesenchymal transition (EMT) through ERK–WNT pathway activation, and CPXM1 may participate in the PI3K–AKT and TGF-β axis signaling pathways to regulate gastric carcinogenesis and progression (18, 19). The level of CPZ expression may influence the prognosis of patients with GC (20), but systematic studies regarding the specific mechanism of CPZ expression, immune regulation-related analysis, and validation of human tissue expression are lacking. The objective of this study is to provide a comprehensive and systematic understanding of the significance of CPZ expression in gastric cancer and its immunoregulatory effects, thereby offering new perspectives and theoretical foundations for identifying molecular biomarkers for gastric cancer diagnosis, developing novel therapeutic targets, and optimizing clinical treatment strategies.

Here, we examined the expression of CPZ in numerous malignancies through The Cancer Genome Atlas (TCGA) and Gene Expression Omnibus (GEO) datasets to focus on analyzing and validating the expression of CPZ in GC, association with the clinical and pathological features of patients with GC, and prognostic implications. Functional enrichment examination of CPZ co-expressed differential genes using R explored potentially relevant biological functions and molecular pathways. The link between the expression of CPZ and the infiltration of fibroblasts and immune cells was investigated in depth, its relationship with immune checkpoints and tumor mutational burden (TMB) was examined, and drug sensitivity was analyzed. Finally, differential CPZ expression in GC tissues and paracancerous tissues was validated using IHC and real-time quantitative PCR (RT-qPCR).

## 2 Materials and methods

### 2.1 CPZ expression analysis in GC

The CPZ gene in pan-cancer was analyzed with the Tumor Immunoassay Resource (TIMER2.0, https://cistrome.shinyapps.io/timer/) (21). We analyzed TCGA RNA sequencing (RNA-Seq) data (https://portal.gdc.cancer.gov/) for GC tissues and adjacent normal tissues with R (https://www.r-project.org/) and the edgeR package (21). We constructed curves for the receiver operating characteristic (ROC) with the timeROC and pROC packages to assess CPZ gene expression differences and their diagnostic significance. CPZ mRNA levels were validated through the database of GEPIA2 (Gene Expression Profiling Interactive Analysis, http://gepia.cancer-pku.cn/) (22) and datasets from Gene Expression Omnibus (GEO): GSE65801 and GSE103236 (https://www.ncbi.nlm.nih.gov/geo/) (23).

### 2.2 Survival analysis according to the expression of CPZ in patients with GC

GEPIA2 is equipped with gene standardization analysis functionality (24), which can classify patients into high and low CPZ expression groups based on the median expression of CPZ, and examine the link between the level of CPZ expression and survival prognosis in patients with GC. TCGA-STAD cases were grouped according to CPZ expression levels (cut-off value: 50%) and clinical information. Survival curves and progression-free survival (PFS) curves were plotted using the R survival, survminer, and rms packages. P < 0.05 indicated statistical significance. The 1-, 3-, and 5-year ROC curves (time-dependent) were constructed to reveal the influence of CPZ expression on the survival prognosis of patients with GC.

### 2.3 Clinical data and prognostic analysis of CPZ in patients with GC

The specific effect of the levels of CPZ expression on survival in patients with GC was assessed using probe ID 210062_S_At from Kaplan-Meier plotter (KM-plotter) (http://kmplot.com/analysis/) (25). The probe data were from the GSE15459 (n = 200), GSE14210 (n = 145), GSE29272 (n = 268), GSE22377 (n = 43), GSE51105 (n = 94), and GSE62254 (n = 300) datasets. CPZ expression levels were categorized into groups of low and high by the optimal cut-offs in the database. We calculated the P-values (log rank), hazard ratios (HR), and the 95% confidence intervals (CI). Furthermore, we generated a three-line table containing the HRs and P-values for the corresponding analyses according to different clinicopathological conditions. Analyses of uni- and multivariate Cox regression were performed with clinical data linked to the expression of CPZ in TCGA-STAD cases using the R survival and ggplot2 packages to identify the association between CPZ expression and the clinical characteristics of patients with GC.

### 2.4 Functional enrichment analysis

Differentially expressed genes (DEGs) closely correlated with CPZ expression in TCGA-STAD samples were determined using the R limma package. The DEG screening criteria were |log2 fold change (FC)| ≥ 1 and corrected P < 0.05. Genes that met the screening criteria were classified as the CPZ high expression group, while genes that did not meet these criteria were classified as the CPZ low expression group. We conducted gene set enrichment analysis (GSEA), Gene Ontology (GO) functional enrichment, and Kyoto Encyclopedia of Genes and Genomes (KEGG) pathway enrichment analyses on the DEGs using the R clusterProfiler and org.Hs.eg.db packages.

### 2.5 Immune infiltration analysis

CPZ gene expression to the tumor-penetrating immune cell ratio in TCGA-STAD samples was assessed using R CIBERSORT. The extent of infiltration of 24 immune cells was visualized using the R Single Sample GSEA (ssGSEA) and GSVA packages. The association between low and high expression of CPZ and immune cell prognosis was examined with the R survival package. The link connecting the expression of CPZ and the levels of fibroblast infiltration in patients with GC was analyzed through the TIMER2.0 immune-gene module.

### 2.6 Correlation analysis of immune checkpoint expression

The correlation between the expression of CPZ and immune checkpoints was explored using Spearman correlation analysis. The results were plotted.

### 2.7 Analysis of tumor mutation load correlation and immunotherapy

Pan-cancer mutation annotation files were obtained from TCGA-STAD database. The tumor mutational burden (TMB) was the total number of somatic mutations per megabase (Mb) (26). The target gene TMB was analyzed using the R maftools package. Immunotherapy analysis related to CPZ expression was performed using the STAD immunophenoscore (IPS) data of The Cancer Imaging Archive (TCIA, http://tcia.at/) database.

### 2.8 Anti-tumor drug sensitivity analysis

Drug data from the Cancer Therapeutics Response Portal (CTRP) and Genomics of Drug Sensitivity in Cancer (GDSC) databases were accessed through the R oncoPredict package. The relationship between CPZ gene expression, drug sensitivity, and the median inhibitory concentration (IC50) in TCGA-STAD samples was analyzed using this package and the Wilcoxon test (cut-off value: 50%).

### 2.9 IHC analysis of CPZ

The expression of CPZ protein in 28 paired GC and paracancerous tissues obtained from The First Affiliated Hospital of Guangxi Medical University was analyzed using IHC. The First Affiliated Hospital of Guangxi Medical University Medical Ethics Committee approved the sample collection and research protocols (2025-E0163). The IHC procedure was as follows: paraffin sections underwent deparaffinization and rehydration, retrieval of antigens, endogenous peroxidase activity blocking, and serum blocking (Blocking solution serum concentration and type: 3% bovine serum albumin (BSA). Dilution buffer: PBS (pH 7.4). Blocking conditions: Block at room temperature for 30 minutes). The sections were incubated with a rabbit polyclonal antibody against CPZ (Catalog No. 15944-1-AP, Sanying, Wuhan, China) at a 1:200 dilution for CPZ IHC staining at 4°C overnight. After secondary antibody incubation, the section accurately reflects the standard IHC staining sequence, where 3,3’-diaminobenzidine (DAB) is used for signal development and hematoxylin serves as the nuclear counterstain. CPZ protein expression levels in immunohistochemical sections were analyzed by average optical density (AOD = cumulative optical density/area) using Fiji. Differential expression of the CPZ protein in different tissues was analyzed using an SPSS 27.0 paired t-test (significance threshold, P< 0.05).

### 2.10 RT-qPCR

Gene expression at the mRNA level, differences in 30 GC and 30 paracancerous fresh tissues obtained from The First Affiliated Hospital of Guangxi Medical University were analyzed using RT-qPCR. Fresh tissue samples from patients with gastric cancer undergoing surgical resection were collected within half an hour after removal from the body, placed in RNA preservation solution, transported in liquid nitrogen, and stored at -80°C in an ultra-low temperature freezer until extraction to prevent RNA degradation.

Total RNA was obtained from the samples and underwent reverse transcription into complementary DNA (cDNA). The specific steps are as follows: 1). mRNA extraction and quality assessment: Place 1 ml of RNA extraction solution (Catalog No. G3013, Servicebio, Wuhan, China) in a grinding tube and pre-cool. Add 5–20 mg of tissue for grinding, then centrifuge at 12000 rpm at 4°C for 10 minutes to collect the supernatant. Add 100 μl of chloroform, centrifuge to separate the aqueous phase, interface, and organic phase. Take 400 μl of the supernatant and add 550 μl of isopropanol to precipitate the RNA (precipitation conditions: -20°C for 15 minutes). RNA Washing and Solubilization: Wash the RNA with 1 ml of 75% ethanol. Add 15 μl of RNA solubilization buffer (Catalog No. T11324, Saint-bio, Shanghai, China) to solubilize the RNA. RNA concentration and purity testing: Take 2.5 μl of the RNA solution to be tested and use a Nanodrop 2000 spectrophotometer (Thermo Fisher Scientific, USA) to test the RNA purity (the A260/A280 ratio should be between 1.8 and 2.0) and concentration. Use 1% agarose gel electrophoresis to verify the integrity of the RNA. Dilute RNA with excessively high concentration to an appropriate ratio to achieve a final concentration of 200 ng/μl. 2). Reverse transcription details: Use 4 μL of SweScript All-in-One RT SuperMix for qPCR (Servicebio, catalog number G3337), 1 μL of gDNA Remover, 10 μL of total RNA, and nuclease-free water to make up to 20 μL. Perform reverse transcription in a 20 μL reaction system. Reaction conditions: 25°C for 5 minutes; 42°C for 30 minutes; 85°C for 5 seconds. Reverse transcription was completed using a standard PCR instrument. 3). qPCR: Take 0.1 ml of PCR reaction plate and prepare the qPCR reaction system (2×Universal Blue SYBR Green qPCR Master Mix 7.5 μl, 2.5 μM gene primers (forward + reverse) 1.5 μl, reverse transcription product (cDNA) 2.0 μl, and Nuclease-Free Water 4.0 μl). Prepare three tubes for each reverse transcription product, seal the tubes, and centrifuge. Set the reaction program: pre-treatment at 95°C for 30 seconds. PCR cycles (40 cycles): 95°C for 15 seconds, denaturation; 60°C for 30 seconds, annealing. Melting curve: 65°C to 95°C, with fluorescence signals collected every 0.5°C increase in temperature. Amplification was performed on a fluorescence quantitative PCR instrument (ETC811, Beijing Dongsheng Innovation Biotechnology Co., Ltd., Beijing, China). GAPDH was the internal reference. The expression stability of GAPDH in the sample set was verified using GeNorm and NormFinder software. The results showed that its coefficient of variation (CV) was <5%, and there was no significant difference between tumor tissue and adjacent tissue (P > 0.05), meeting the selection criteria for internal reference genes. The CPZ primer sequences were as follows: 5′-GCATTCGCCACGACATCA-3′ (forward) and 5′-CTCCGCAGCCCATGAATAAA-3′ (reverse). The relative expression of mRNA was quantified using the comparative cycle threshold [2(-ΔΔCt)] method. Use the mRNA expression levels of adjacent normal tissue as a calibrator (in ΔΔCt calculations, the calibrator group ΔCt = 0). The expression data analysis was conducted with the SPSS 27.0 Wilcoxon signed-rank test (significance threshold, P< 0.05).

## 3 Results

### 3.1 Differential expression of CPZ in GC and its diagnostic value

**Figure 1:**
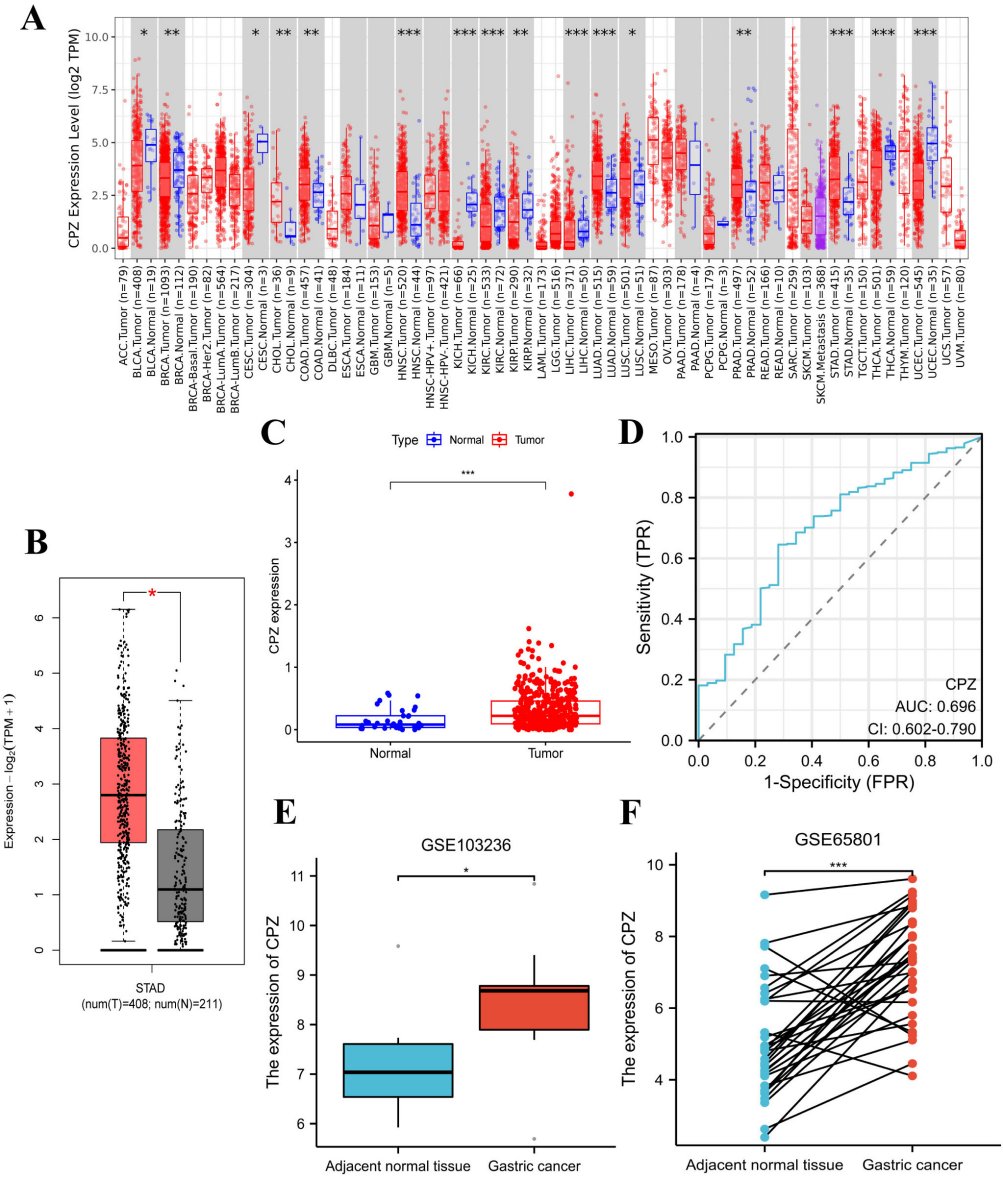
Differences in the expression of CPZ between Gastric Cancer (GC) and normal tissues and their STAD diagnostic value. **(A)** Differences in CPZ expression in cancerous tissues and normal tissues of different cancers in the TIMER2.0 database. **(B)** Differential expression of CPZ in GC and normal tissues based on the GEPIA2 database. **(C)** Differential expression of CPZ in GC and normal tissues based on TCGA-STAD. **(D)** ROC diagnostic curve of CPZ expression in TCGA-STAD database. **(E)** Differential expression of CPZ in GC and adjacent normal tissues in the GSE103236 dataset. **(F)** Pair-wise analysis of 32 GC and paracancerous normal tissue pairs in the GSE65801 dataset. *P < 0.05, **P < 0.01, ***P < 0.001, ****P < 0.0001.

The TIMER2.0 analysis of pan-cancer identified significant CPZ overexpression in tissues from GC compared to normal tissues (**Figure 1A**). The GEPIA2 analysis of 619 specimens (GC tissues, n = 408; normal tissues, n = 211) demonstrated high CPZ expression in GC tissues (**Figure 1B**). This result was validated by analyzing RNA-Seq data from 448 specimens (normal, n = 36; patients with GC, n = 412) in the TCGA-STAD dataset using R. The results indicated that tissues from GC had significantly higher expression of CPZ than normal tissues (**Figure 1C**). From the ROC curve analysis, the area under the curve (AUC) was 0.696 (95% CI: 0.602–0.790), indicating a potential reference value for distinguishing between normal and STAD tissues (**Figure 1D**). As TIMER2.0, GEPIA2, and TCGA contain disease data from foreign populations, and the Chinese population has specific disease characteristics, we conducted differential expression analysis using the GEO datasets GSE103236 (containing nine specimens each from GC and normal gastric tissue) and GSE65801 (containing 32 specimens each from GC and normal gastric tissue), which mainly include Chinese populations. These analyses confirmed significantly upregulated expression of CPZ in GC tissues (**Figures 1E, F**).

### 3.2 Survival analysis according to the expression of CPZ in patients with GC

**Figure 2:**
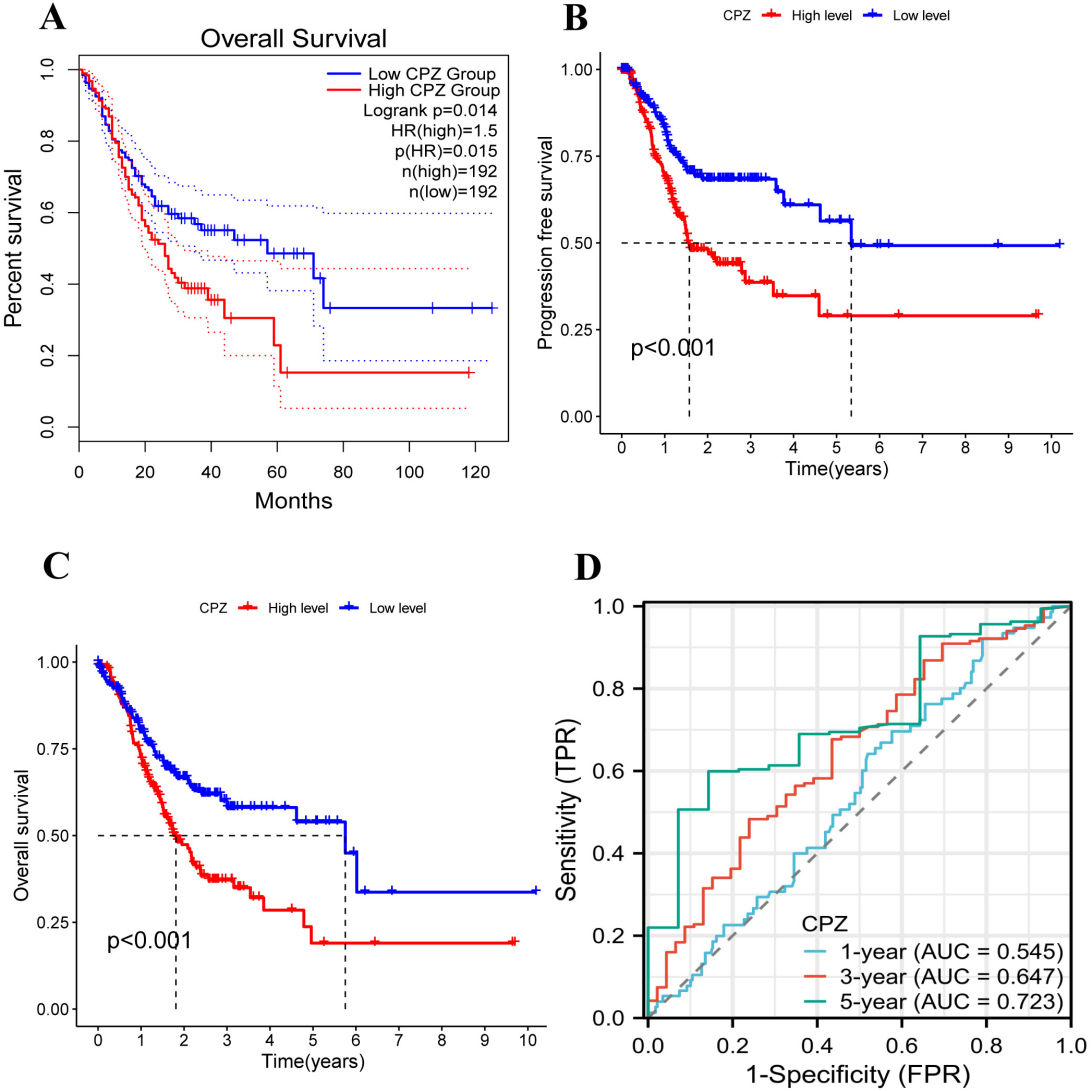
Analysis of the survival and prognosis of patients with GC with low and high expression of CPZ. **(A)** Overall Survival (OS) of GC patients with low and high expression of CPZ according to the database of GEPIA2. **(B)** OS curves of patients with GC with low and high expression of CPZ according to TCGA-STAD. **(C)** Progression Free Survival (PFS) curves of patients with GC with low and high expression of CPZ based on TCGA-STAD. **(D)** Receiver Operating Characteristic (ROC) curves for CPZ expression in GC patients at 1, 3, and 5 years using TCGA-STAD data.

Based on the high expression of CPZ in the tissues from GC, we examined the influence of CPZ on the survival of patients with GC. The GEPIA2 database analysis revealed a significant association between elevated expression of CPZ and poorer overall survival (OS) in patients with GC (P < 0.05) (**Figure 2A**). Due to the differences in clinical information from the different databases, we validated the results using TCGA-STAD clinical data to conclude that the median low-CPZ expression group had significantly higher OS and PFS than the high-expression group (P < 0.001) (**Figures 2B, C**). Furthermore, a ROC analysis dependent on time was conducted to assess the dynamics of CPZ in the prognosis of patients with STAD more comprehensively, and demonstrated that the CPZ value in predicting the prognosis of patients with STAD gradually increased with time (ROC curve AUC for years 1, 3, and 5 = 0.545, 0.647, and 0.723, respectively) (**Figure 2D**). Collectively, the findings indicated that high expression of CPZ in patients with GC was linked to poor prognosis and that CPZ may be a biological marker for forecasting long-term prognosis in GC.

### 3.3 Clinical correlation and prognostic importance of the expression of CPZ in patients with GC

**Figure 3:**
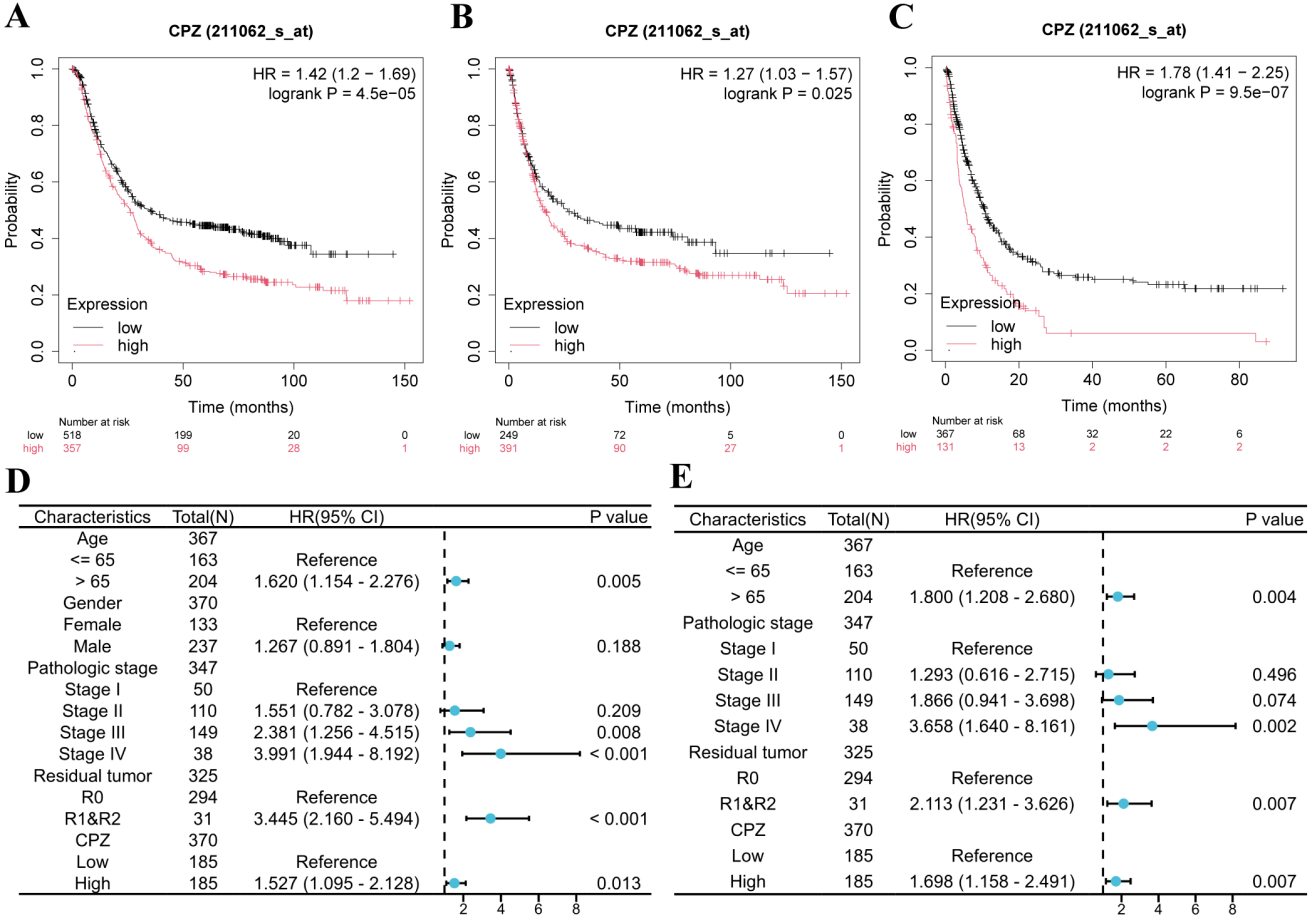
Kaplan-Meier survival analysis and Cox analysis of patients with Gastric Cancer (GC) with low and high expression of CPZ. **(A–C)** Patients’ Kaplan-Meier survival curves: **(A)** Overall Survival (OS), **(B)** Progression Free (PF), **(C)** Post Progression Survival (PPS). **(D)** Forest plot of HR and P-value of Cox univariate analysis of the expression of CPZ according to gender, age, pathological stage, and tumor residue in patients with GC. **(E)** Forest plot of HR and P-value of Cox multivariate analysis of CPZ expression versus age, pathological stage, and tumor residue in patients with GC.

Based on analyzing the differential expression of CPZ in GC and survival prognosis, we explored the correlation between CPZ expression and the prognostic clinical characteristics of patients with GC through KM-plotter. High CPZ expression was significantly associated with poorer survival prognosis for PFS, OS, and Post-Progression Survival (PPS) (**Figures 3A–C**), which was consistent with our previous findings. We also analyzed the relationship between the expression of CPZ and the prognosis and characteristics of the tumor. There was a significant association between the expression of CPZ and stage 3, T4 stage, N1 and N2 stage, and M0 stage disease; intestinal and diffuse Lauren classifications; moderate differentiation; gender; and poorer OS and PF (**Table 1**). The analysis indicated that the N stage had the highest overall HR among the significant correlates, which suggested that the expression of CPZ was linked to the degree of lymph node metastasis, which affects the prognosis of patients with GC.

**Table 1:** Effect of CPZ on clinical prognosis of patients with GC under different clinicopathologic features.

Clinicopathological characteristics	OS (n = 875)			PFS (n = 640)		
	N	HR	P-value	N	HR	P-value
Stage						
1	67	2.92 (0.83–10.29)	0.082	60	2.17 (0.59–7.96)	0.23
2	140	1.76 (0.95–3.24)	0.068	131	1.28 (0.7–2.36)	0.42
3	305	1.70 (1.26–2.28)	0.0004	186	1.72 (1.19–2.49)	0.0038
4	148	1.74 (1.18–2.56)	0.0043	141	0.84 (0.56–1.27)	0.41
T stage						
2	241	1.97 (1.27–3.06)	0.002	239	1.46 (0.96–2.23)	0.075
3	204	1.37 (0.95–1.97)	0.088	204	1.24 (0.86–1.77)	0.24
4	38	2.51 (1.07–5.89)	0.029	39	2.22 (1.01–4.85)	0.041
N stage						
0	74	2.52 (0.93–6.83)	0.059	72	2.43 (0.9–6.58)	0.07
1	225	2.19 (1.45–3.32)	0.0001	222	1.85 (1.25–2.73)	0.0019
2	121	1.95 (1.23–3.09)	0.0038	125	1.79 (1.15–2.77)	0.0089
3	76	1.48 (0.82–2.65)	0.19	76	1.36 (0.77–2.41)	0.29
M stage						
0	444	1.73 (1.31–2.29)	8.7 × 10^-5^	443	1.57 (1.19–2.06)	0.0011
1	56	1.54 (0.87–2.75)	0.14	56	0.43 (0.23–0.79)	0.0053
Lauren classification						
Intestinal	320	1.92 (1.4–2.63)	3.8 × 10^-5^	263	1.53 (1.07–2.18)	0.019
Diffuse	241	1.57 (1.11–2.22)	0.0094	231	1.56 (1.11–2.2)	0.0099
Mixed	32	2.61 (0.92–7.39)	0.061	28	1.79 (0.4–7.95)	0.44
Differentiation						
Poor	165	0.86 (0.56–1.31)	0.49	121	0.79 (0.49–1.27)	0.33
Moderate	67	2.01 (1.02–3.94)	0.04	67	2.03 (1.06–3.89)	0.03
Gender						
Female	236	1.78 (1.17–2.73)	0.0069	201	1.76 (1.13–2.75)	0.012
Male	544	1.57 (1.26–1.95)	4.1 × 10^-5^	437	1.33 (1.03–1.72)	0.026

We assessed the clinical prognostic importance of CPZ in GC through analyses of uni- and multivariate Cox regression. The univariate Cox regression analysis demonstrated that high CPZ expression (HR = 1.527), age > 65 years (HR = 1.620), stage III (HR = 2.381) and IV (HR = 3.991), and tumor microscopic residue (R1) and naked eye residue (R2) (HR = 3.445) were significantly associated with OS in STAD cases (P < 0.05) (**Figure 3D**). Under multivariate Cox regression analysis, high CPZ expression (HR = 1.698), age > 65 years (HR = 1.800), stage III (HR = 1.866) and IV (HR = 3.658), and R1 and R2 (HR = 2.113) were independent prognostic risk factors for patients with STAD (**Figure 3E**), significantly affecting their prognosis. The results suggest that high expression of CPZ is vital for determining a poor prognosis in patients with GC and may provide new avenues for diagnostic and therapeutic protocols and prognostic monitoring of such patients.

### 3.4 Prediction of CPZ-related functions in GCs

**Figure 4:**
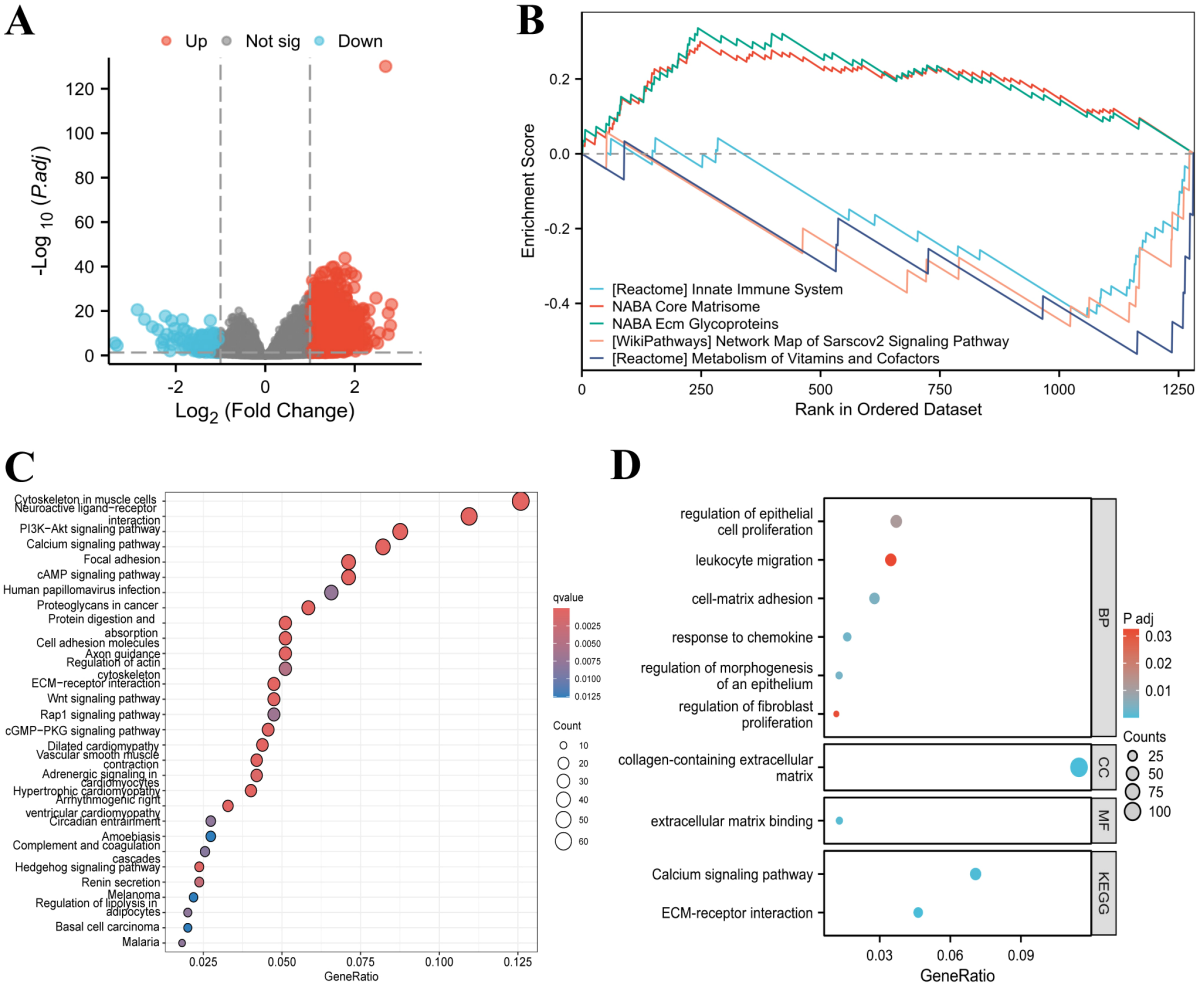
Differentially Expressed Gene (DEG) functional analysis. **(A)** Volcano map of DEGs identified according to TCGA-STAD expression of CPZ. **(B)** Gene Set Enrichment Analysis (GSEA) analysis of DEGs, showing upregulation of DEGs in the innate immune system, NABA Core Matrisom, and genes encoding structural ECM glycoproteins. **(C)** Gene Ontology (GO) enrichment analysis of DEGs with high CPZ expression. **(D)** DEG enrichment of enrichment in Biological Processes (BP), Cellular Components (CC), Molecular Function (MF), and enrichment of Kyoto Encyclopedia of Genes and Genomes (KEGG).

The samples were divided into groups of low and high expression of CPZ according to TCGA-STAD differential CPZ expression (|log2 fold change (FC)| ≥ 1 and corrected P < 0.05), which revealed 1776 DEGs (1594 upregulated genes and 182 downregulated genes) (**Figure 4A**). These DEGs underwent GSEA and GO/KEGG functional enrichment analyses. GSEA enrichment analysis is associated with the entire set of DEGs. GSEA revealed an association between the DEGs and immune processes and cellular biological functions. The related enriched pathways in the innate immune system, genes encoding the core ECM, and genes encoding structural ECM glycoproteins, showed upregulated activity in co-expression with CPZ high expression (**Figure 4B**).

The GO/KEGG analyses of enrichment in pathways demonstrated that genes highly expressed in CPZ were significantly enriched in functional pathways such as cytoskeleton in muscle cells; neuroactive ligand–receptor interaction; the signaling pathways of PI3K–Akt, calcium, cAMP; focal adhesion; and proteoglycans in cancer (**Figure 4C**). There were significant CPZ-highly expressed genes enrichment in biological processes (BP) (regulation of epithelial cell proliferation, leukocyte migration, adhesion of cell–matrix, chemokine response, epithelial morphogenesis regulation, regulation of fibroblast proliferation). In molecular functions (MF) and cellular components (CC), the CPZ-highly expressed genes were significantly enriched in ECM binding and collagen-containing ECM, respectively. The main KEGG pathways identified for the related DEGs included the calcium signaling pathway and ECM receptor interaction (**Figure 4D**). The enrichment analyses indicated that CPZ may participate in GC development and progression through various signaling pathways and the ECM, which warrants further in-depth investigation.

### 3.5 Link between CPZ and immune infiltration in GC

**Figure 5:**
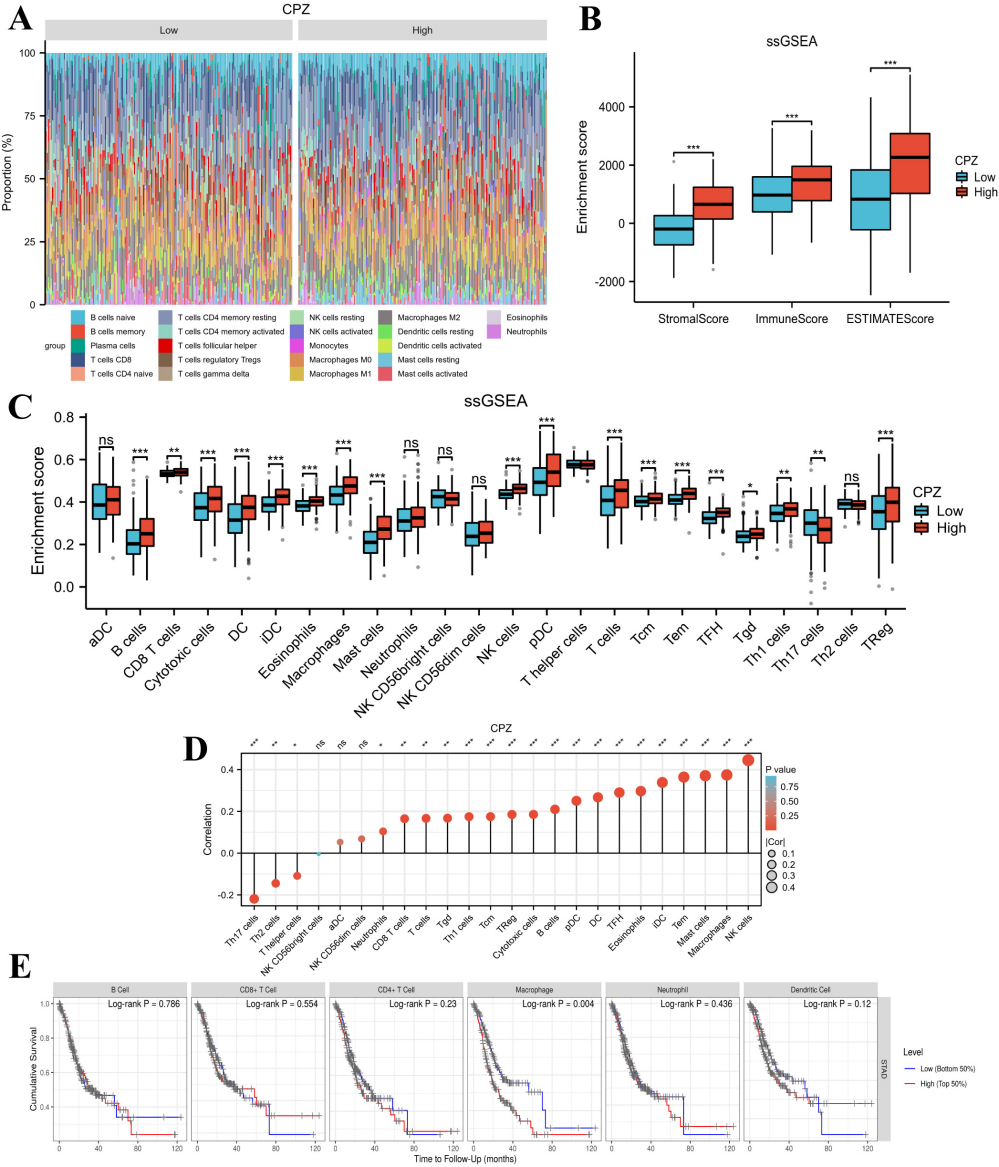
Association connecting CPZ and infiltration of immune cells. **(A)** Infiltrating immune subpopulation proportions in Stomach Adenocarcinoma (STAD). **(B)** Link between immune infiltration score and expression of CPZ based on the ESTIMATE score and immune and stromal scores. **(C)** Differing infiltration of immune cells based on the expression of CPZ. **(D)** Analysis of the correlation between the expression of CPZ and immune cells. Positive correlation, Cor > 0; negative correlation, Cor < 0. **(E)** CPZ expression in STAD and immune cell prognosis analysis. *P < 0.05, **P < 0.01, ***P < 0.001.

As the DEG functional enrichment analysis suggested CPZ involvement in immune infiltration pathways, we analyzed the relationship between the expression of CPZ and immune cell subset infiltration in STAD through CIBERSORT (**Figure 5A**). The analysis revealed that the group with high CPZ expression had significantly higher stromal, immune, and ESTIMATE scores than the group with low expression (**Figure 5B**), indicating a high immune and stromal cell content and low tumor purity. Interestingly, ssGSEA suggested increased immune cell subset infiltration in the high expression of CPZ group, including B cells, CD8+ T cells, cytotoxic cells, dendritic cells, immature dendritic cells, eosinophils, macrophages, mast cells, natural killer (NK) cells, plasmacytoid dendritic cells, helper T (Th) cells, T cells, central memory T cells, effector memory T cells, T follicular helper cells, γδT cells, Th1 cells, and regulatory T cells (**Figure 5C**).

Correlation analysis of CPZ gene expression and immune cell infiltration also demonstrated that CPZ expression was associated with neutrophils, CD8+ T cells, T cells, γδT cells, Th1 cells, regulatory T cells, central memory T cells, cytotoxic cells, B cells, plasma cell-like dendritic cells, T follicular helper cells, dendritic cells, eosinophils, immature dendritic cells, effector memory T cells, mast cells, and macrophages, and was positively correlated with NK cell infiltration. CPZ expression was correlated negatively with Th17, Th2, and Th cell numbers (**Figure 5D**). Subsequently, we explored the prognostic significance of the expression of CPZ and infiltration by immune cells. The results demonstrated that high expression of CPZ and macrophage infiltration affected the OS in patients with GC (P < 0.05) (**Figure 5E**).

We used TIMER2.0 to investigate the link connecting the infiltration levels of cancer-associated fibroblasts (CAFs) and CPZ gene expression across TCGA cancer datasets. The analysis identified a significant positive association connecting CPZ expression and CAF infiltration in GC, cholangiocarcinoma (CHOL), and breast cancer (BC) (**Supplementary Figure 1A**). Different algorithms revealed a significant positive correlation of fibroblast infiltration in GC (**Supplementary Figure 1B**).

### 3.6 CPZ expression and immune checkpoints in GC

**Figure 6:**
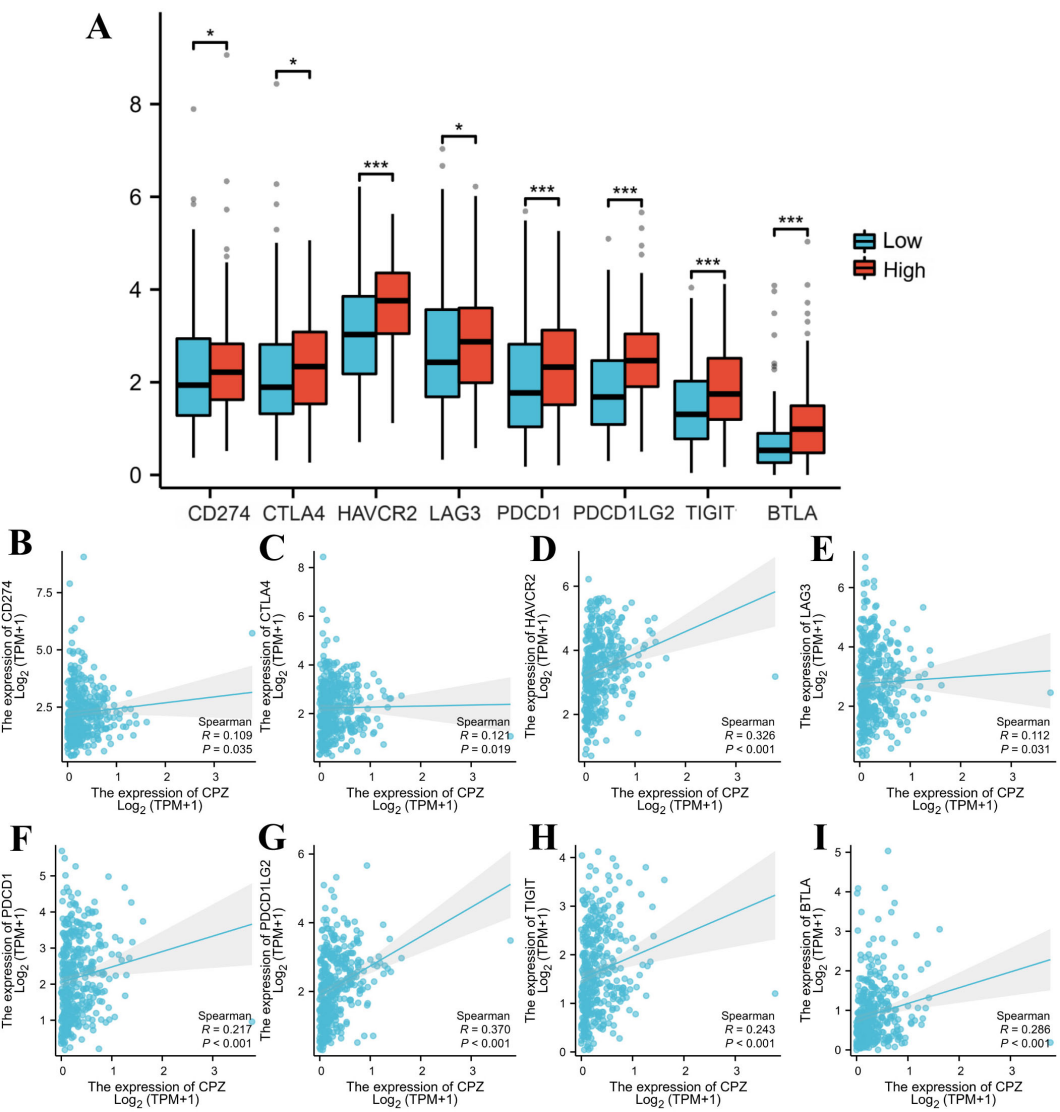
Identification of immune checkpoints associated with CPZ in Gastric Cancer (GC). **(A)** Comparison of immune checkpoint expression based on the differential expression of CPZ. **(B–I)** Spearman correlation analysis between the expression of CPZ and CD274 **(B)**, CTLA4 **(C)**, HAVCR2 **(D)**, LAG3 **(E)**, PDCD1 **(F)**, PDCD1LG2 **(G)**, TIGIT **(H)**, and BTLA **(I)**. *P < 0.05, **P < 0.01, ***P < 0.001.

As GC progression is closely related to the immune microenvironment, we performed Spearman correlation analysis of the immune checkpoints and target genes to investigate the association between CPZ and the immune microenvironment. There was a significant positive correlation connecting CPZ high expression with common immune checkpoints, including cluster of differentiation 274 (CD274), lymphocyte-activation gene 3 (LAG3), hepatitis A virus cellular receptor 2 (HAVCR2), CTLA4, programmed cell death protein 1 (PDCD1), TIGIT, PDCD1 ligand 2 (PDCD1LG2), and B and T lymphocyte attenuator (BTLA) (**Figures 6A–I**).

### 3.7 CPZ expression correlated with TMB and immunotherapy response in GC

**Figure 7:**
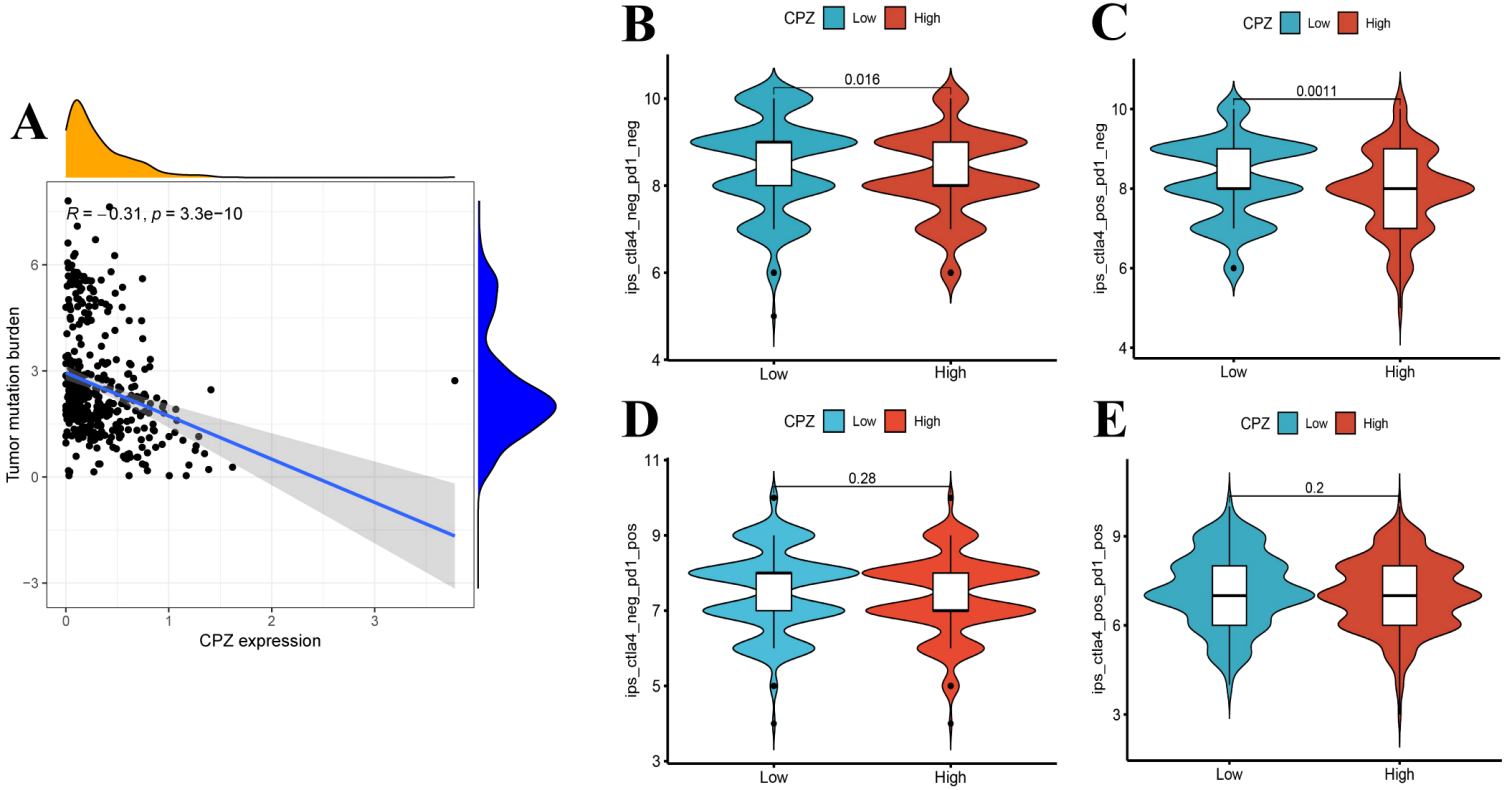
Analysis of Tumor Mutation Burden (TMB) and Immunophenotypic Score (IPS) under CPZ expression in Gastric Cancer (GC). **(A)** Correlation analysis of CPZ expression with TMB based on TCGA-STAD. **(B–E)** IPS of CPZ expression in GC under the following immunophenotypes: **(B)** CTLA4_neg+PD1_neg, **(C)** CTLA4_pos+PD1_neg, **(D)** CTLA4_pos+PD1_pos, and **(E)** CTLA4_neg+PD1_pos.

We confirmed that CPZ gene expression can influence the GC immune microenvironment, and TMB correlation analysis revealed a significant negative correlation between the expression of CPZ and TMB (**Figure 7A**). This result indicated that high CPZ gene expression in GC somewhat inhibits tumor mutation burden and aids in maintaining tumor cell genome stability.

Immunotherapy analysis demonstrated that in samples that were CTLA4-negative (CTLA4_neg) and PD1-negative (PD1_neg), the group with low expression demonstrated significantly higher IPS compared to the group with high expression (**Figures 7B, C**), suggesting that patients with GC with low CPZ expression may obtain better immunotherapy results. In samples that were CTLA4-positive (CTLA4_pos) and PD1_neg, the CPZ low-expression group had a higher IPS and a better response when receiving anti-CTLA4 immunotherapy. Notably, the PD1-positive (PD1_pos) samples between the groups of low and high expression did not have a significantly different IPS (**Figures 7D, E**), suggesting that CPZ only is insufficient to influence the GC immune response under these conditions. However, PD1_pos suggested that patients with GC have a certain potential for response to immune checkpoint inhibitors, and clinical treatment should combine other immune targets to enable a comprehensive evaluation.

### 3.8 Differential CPZ expression and anti-tumor drug sensitivity in patients with GC

**Figure 8:**
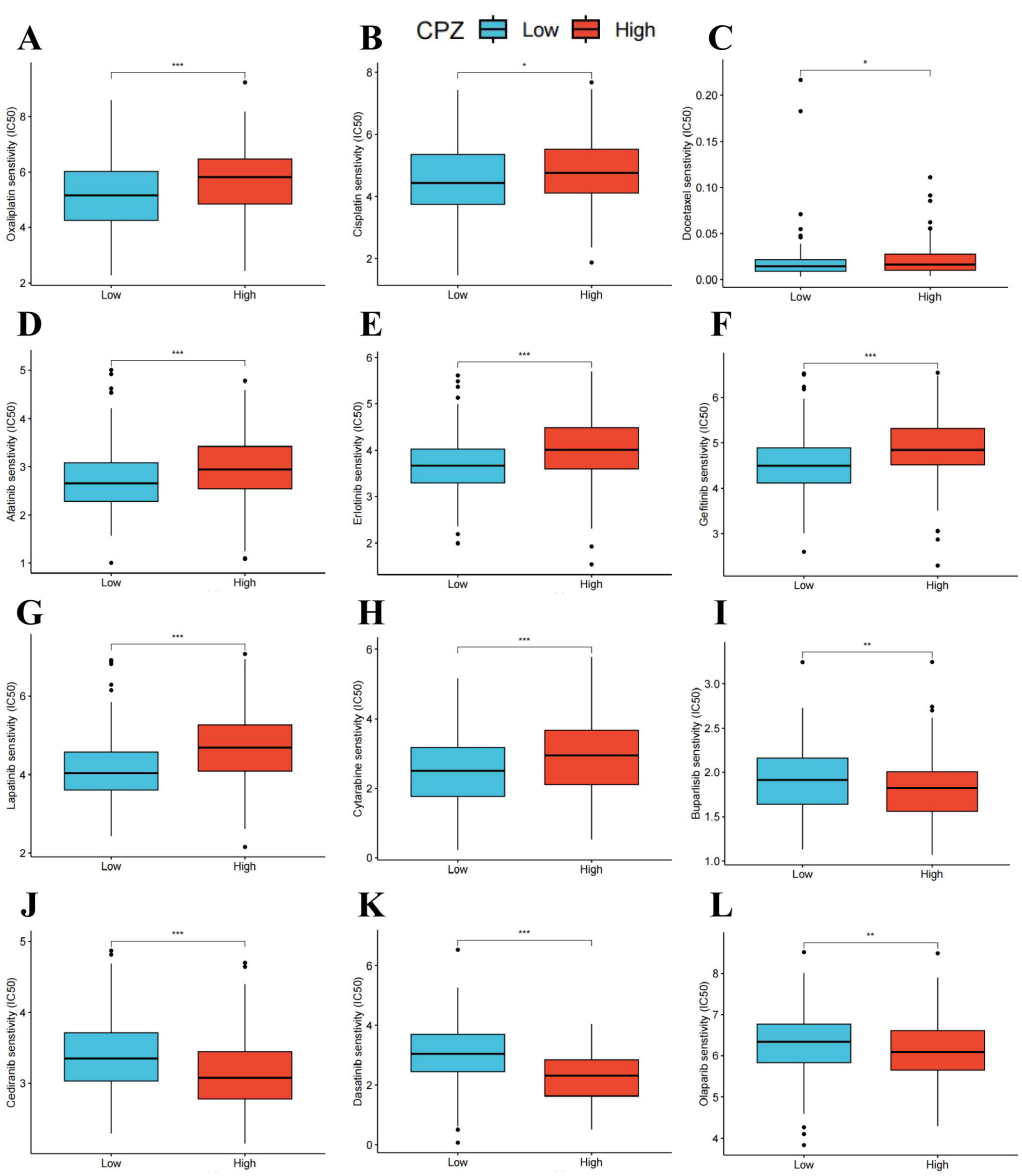
Drug sensitivity of CPZ in Stomach Adenocarcinoma (STAD). **(A–L)** The predicted Half maximal Inhibitory Concentration (IC50) of oxaliplatin **(A)**, cisplatin **(B)**, docetaxel **(C)**, afatinib **(D)**, erlotinib **(E)**, gefitinib **(F)**, lapatinib **(G)**, cytarabine **(H)**, buparlisib **(I)**, cediranib **(J)**, dasatinib **(K)**, and olaparib **(L)** based on CPZ expression. *P < 0.05, **P < 0.01, ***P < 0.001.

Chemotherapeutic drugs are one of the main methods for treating GC. However, drug toxicity and drug resistance often limit chemotherapy efficacy. The relationship between CPZ gene expression and drugs related to cancer treatment was investigated by dividing the cases into groups based on CPZ expression (cut-off value: 50%). Drug sensitivity was analyzed using the IC50. The analysis and visualization of the data indicated that some anti-tumor drugs had significantly higher IC50 values in the group with high expression of CPZ than in the group with low expression of CPZ, such as oxaliplatin, cisplatin, docetaxel, afatinib, erlotinib, gefitinib, lapatinib, and cytarabine (**Figures 8A–H**). The analysis suggested that patients with STAD with high expression of CPZ might benefit less from chemotherapy and targeted therapy compared to those with low CPZ expression, indicating a certain degree of drug resistance. Conversely, some drugs (buparlisib, cediranib, dasatinib, olaparib) had lower IC50 values in the group with high expression of CPZ than in the group with low expression of CPZ (**Figures 8I–L**), suggesting that high CPZ expression sensitizes GC cells to these drugs. The drug sensitivity analysis indicated that high CPZ expression may render GC partially resistant to common drugs such as oxaliplatin, cisplatin, and docetaxel. However, altered CPZ expression did not affect the sensitivity to common GC chemotherapeutic drugs such as paclitaxel, tegafur-gimeracil-oteracil potassium, capecitabine, and fluorouracil. These results provide a certain chemotherapeutic basis for developing personalized treatment approaches for clinical patients with GC.

### 3.9 Upregulation of CPZ in human GC tissues

**Figure 9:**
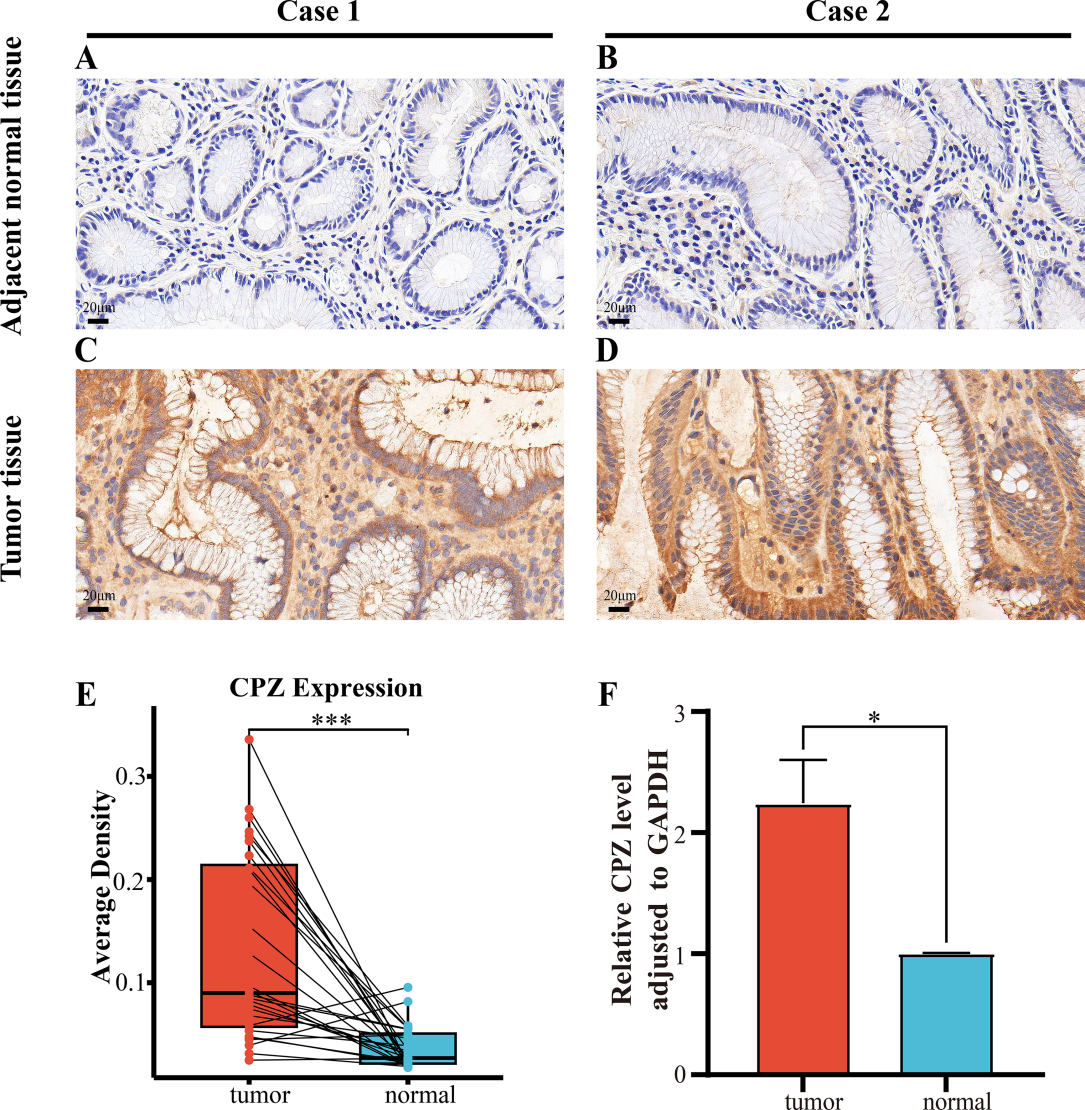
IHC and RT-qPCR of CPZ overexpression in human Gastric Cancer (GC) tissues compared with contiguous non-cancerous tissues. **(A, B)** IHC images of CPZ expression in contiguous non-cancerous tissues (×400 magnification). **(C, D)** IHC images of the expression of CPZ in GC tissues (×400 magnification). **(E)** Semi-quantitative analysis comparison of the expression of CPZ protein in GC and contiguous non-cancerous tissues (AOD values). **(F)** RT-qPCR comparison of the levels of CPZ expression in GC and adjacent non-cancerous tissues. *P < 0.05, ***P < 0.001.

The previous bioinformatics analysis results through *in vitro* experiments were validated through IHC staining of 29 paired fresh GC and adjacent normal tissues to compare CPZ expression and distribution in the tissues. CPZ expression levels were significantly higher (**Figure 9E**) (P < 0.001) in GC tissues (**Figures 9C, D**) than in the adjacent normal tissues (P < 0.001) (**Figures 9A, B**). Analysis of RNA expression in 30 fresh GC and contiguous non-cancerous tissues revealed significantly higher expression of CPZ mRNA in the GC tissues (**Figure 9F**).

## 4 Discussion

The metallocarboxypeptidase gene family is closely associated with the infiltration and metastasis of various tumors, including pancreatic cancer, lung adenocarcinoma (15), and GC (18, 19). Researchers found that inhibiting CPE expression in pancreatic tumor cells can suppress cell growth and metastasis by regulating pathways such as p53, Wnt signaling, and downstream target nuclear factor κB (NF-κB), thereby increasing the drug sensitivity of pancreatic cancer patients to cisplatin (27, 28). Silent CPE expression can suppress cyclin D1 expression, leading to cell cycle arrest and inhibiting the proliferation and metastasis of osteosarcoma cells (29). Inhibiting Carboxypeptidase A6 (CPA6) expression in colorectal cancer can suppress Akt/mTOR signaling activation and inhibit tumor growth (30). CPZ is present in the extracellular matrix and is a member of the carboxypeptidase family. It exhibits distinct immunohistochemical staining in human colon cancer (12) and pituitary adenoma (17), providing evidence for its expression in tumors. Furthermore, significantly reduced methylation of the 5’ UTR of CPZ on chromosome 4p16 and increased CPZ gene expression in neuroblastoma patients may increase susceptibility to neuroblastoma (13). We conducted an in-depth investigation into the expression of CPZ in GC and its clinical implications. Compared with normal tissues, the GC tissues had significantly higher expression of CPZ. Patients with GC with high expression of CPZ had lower survival rates that became more pronounced with time, and CPZ may be a relatively reliable diagnostic and prognostic indicator. Clinicopathological characterization identified a significant association connecting high CPZ expression and prognosis related to pathological stage, lymph node metastasis type, and Lauren classification, particularly in stage 3, N1 stage, and N2 stage, indicating that CPZ expression may promote lymph node metastasis to affect prognosis. The uni- and multivariate analyses demonstrated that CPZ was an independent GC prognostic risk factor.

GSEA and GO/KEGG functional enrichment analyses of the DEGs co-expressed with CPZ in GC indicated that CPZ is involved in GC immune infiltration regulatory pathways, calcium signaling, cell–matrix interactions, co-factor metabolism, and the PI3K–Akt signaling pathway. Metal ion-induced apoptosis is closely related to tumor progression. Tu et al. focused on the gene characteristics of lncRNA related to cuproptosis in gastric adenocarcinoma, constructed a prognostic model, and evaluated the efficacy of anticancer drugs (31). Calcium signaling is an important intracellular signal process mainly regulated by key factors such as calcium ion influx, endoplasmic reticulum calcium release, cytoplasmic calcium-binding proteins, calcium pump regulation and transport, calcium-modulating enzymes, and second messengers (IP3, cADPR). Abnormalities in the function and components of this pathway are closely related to tumor proliferation, metastasis, apoptosis, and gene mutation (32). Researchers have confirmed that xanthone dimers (Xds) significantly increase intracellular Ca²^+^ concentration by specifically activating the reverse transport mode of sodium-calcium exchanger 1 (NCX1), thereby inhibiting the downstream PI3K/AKT/β-catenin signaling pathway and ultimately leading to the death of gastric cancer cells (33). TRPV6 is a calcium channel that is overexpressed in various cancers, and its inhibitors can lead to better treatment outcomes in patients with colorectal, pancreatic, and ovarian cancer (34). Regulation of calcium ions and their channels is helpful in cancer treatment. CPZ may affect GC progression by influencing calcium ion transport regulation in the calcium signal pathway. Therefore, in subsequent pathway mechanism studies, we can start with the functional expression of CPZ in GC.

The examination of the infiltration of immune cells and CPZ expression revealed that high CPZ expression promoted the increase of stromal and immune cells somewhat, resulting in an overall decrease in the proportion of tumor cell components, which means that CPZ is important in tumor microenvironment regulation. In the tumor microenvironment, adaptive immune cells, myeloid immune cells, stromal components, and vascular components constitute a complex tumor ecosystem. CD8+ T cells can explicitly identify and bind to the T cell receptor (TCR) on the cancer cell surface and destroy target cells by mediating apoptosis or FASL–FAS-mediated cell death (35, 36). B cell-mediated humoral immunity has a dual role: it combats tumors by activating complement and cell-mediated cytotoxicity dependent on antibodies, and promotes inflammation and inhibits immunity by secreting inflammatory factors and immune complexes, promoting tumor growth (37, 38). The Th1 subtype of CD4+ T cells (Th cells) can produce interferon gamma (IFNγ) and TNF-α to directly kill cancer cells and can exert anti-tumor effects by aiding B cells and CD8+ cells (39, 40). Th17 cells can produce IL-17 to promote inflammatory responses and angiogenesis, enhancing the immune adaptability of the tumor microenvironment (41, 42). We determined that CPZ increased T cell, cytotoxic cell, B cell, CD8+ T cell, macrophage, dendritic cell, and mast cell infiltration. Macrophage infiltration in GC affects patient survival prognosis. Additionally, high CPZ expression inhibits Th17, Th2, and Th cell infiltration in GC, indicating that CPZ expression decreases the killing ability of some immune cells.

Macrophages in GC can induce mesenchymal stem cell (MSC) transformation into fibroblasts (43). CAFs can synthesize and remodel the ECM, altering the cell arrangement and affecting immune cell behavior, with considerable immunomodulatory effects that favor tumor immune evasion (44, 45). Our analysis indicated that higher expression of CPZ in GC tissues is accompanied by a higher fibroblast infiltration level. Trastuzumab, a first-line drug targeting HER2 in advanced GC, can target and bind HER2 extracellular structural domain 4, inhibiting the activation of signals downstream and the proliferation of cancer cells. However, trastuzumab treatment effects are not ideal in some patients with advanced HER2-positive GC, which may be related to intratumoral HER2 heterogeneity (46, 47), loss of HER2 expression after treatment (48), gene amplification mutations (49, 50), and abnormal intracellular signaling (RTK–RAS–PI3K) (51). GC has far fewer therapeutic targets than other cancers. Therefore, identifying new targets related to anti-tumor drugs is extremely important for GC treatment. CPZ participates in adaptive immune responses and affects the TME, has excellent immunomodulatory capabilities, and promotes cancer development. This means that CPZ is a prognostic influencing molecule in GC and a possible biological target for GC therapy.

Immune checkpoints, a series of immunosuppressive molecules, are expressed by immune cells to control immune cell secretion factors and modulate immune function (52). Tumor cells in the tumor microenvironment can activate immune checkpoint functions to inhibit T cell antigen presentation in immunity of the tumor, which enables immunity escape and survival by tumor cells (53). GC treatment widely involves immune checkpoint inhibitors (ICIs). Immunosuppressive therapies targeting PD1 and PD ligand 1 (PD-L1) binding to initiate immune regulation can improve survival rates in patients with advanced disease (54, 55). PD1 inhibitors such as sintilimab (ORIENT-16) (56, 57) and pembrolizumab (KEYNOTE-859) (58) combined with standard chemotherapy can enhance the survival prognosis in advanced HER2-negative GC cases. Ipilimumab is a CTLA4 inhibitor that can restore and induce T cell-mediated tumor cell killing (59). However, in clinical practice, most patients develop drug resistance after long-term treatment with a certain immunosuppressant, leading to immune tolerance. We determined that the expression levels of several immune checkpoints (CD274, CTLA4, HAVCR2, LAG3, PDCD1, PDCD1LG2, TIGIT, BTLA) increased with high expression of CPZ, suggesting that cases with high CPZ expression have more immune blockade targets to approach, allowing them to benefit from it. We also determined that a higher TMB in GC is accompanied by lower CPZ expression. A TMB that is high facilitates tumor microenvironment neoantigen production, and the increased immunogenicity of the neoantigens enhances immune reactivity and improves the therapeutic effect of ICIs (60). However, high CPZ expression does not have a therapeutic advantage in this regard. Our immunotherapy analysis determined that low-CPZ expression patients responded better to CTLA4 blockade. Nevertheless, more clinical trials are needed to confirm the efficacy of other immunostimulatory on CPZ expression.

Systemic intravenous chemotherapy is a necessary component in treating advanced GC. Our exploration of the link between CPZ expression and the sensitivity to numerous anti-tumor drugs revealed that high CPZ expression is associated with reduced sensitivity to some first-line chemotherapeutic drugs, such as oxaliplatin, cisplatin, and docetaxel. Nonetheless, the sensitivity to individual chemotherapeutic drugs, such as dasatinib, increases association with high CPZ expression, while CPZ expression does not affect the efficacy of other drugs. Dasatinib is a second-generation adenosine triphosphate (ATP)-competitive protein tyrosine kinase inhibitor that can induce senescent cell apoptosis by inhibiting the family of non-receptor tyrosine kinases (Src) (61). Src is involved in cell proliferation, migration, and tumor growth, and its high activation is closely related to GC (62, 63). Dasatinib increases GC sensitivity to cisplatin and improves chemotherapy efficacy (64), which can point us in the right direction for our subsequent drug target research. Drug sensitivity analysis of tumors can provide targeted chemotherapy treatment strategies for clinical patients expressing CPZ.

As bioinformatics analysis results are not always reliable, we collected samples from clinical patients with GC and confirmed statistically significant increased CPZ expression in their tumor tissues through IHC and RT-qPCR. This conclusion emphasizes the importance of CPZ as a predictive marker of immune-related biological prediction and prognosis in GC. We analyzed the prognostic and immunomodulatory predictive potential of CPZ in GC systematically. However, this study was subject to limitations. First, the observed correlations between CPZ expression and immune checkpoint molecules do not establish causality, and functional experiments are needed to explore CPZ’s mechanistic role in immune regulation (e.g., gene knockdown/overexpression, pathway assays to explore its biological function). Second, immune-related analyses were based on bulk RNA-seq data, which do not account for the complexity of the tumor microenvironment or cell-type-specific expression patterns (stromal vs tumor vs immune) that single-cell RNA-seq data could provide. Third, the sample size for the clinical validation in this study is limited and primarily derived from a single cohort. It is necessary to actively collaborate with multi-center research teams to conduct broader validation across different patient cohorts to confirm the universality of CPZ as a biomarker. Nevertheless, high CPZ expression in patients with GC is a therapeutic perspective worth exploring in depth.

## 5 Conclusion

CPZ expression is high in GC and is a valuable diagnostic prognostic factor. CPZ may be a crucial target for treating GC. High CPZ expression modulates the expression of immune checkpoints and immune cell infiltration through multiple mechanisms, promoting GC progression.

## Data Availability

The original contributions presented in the study are included in the article/**Supplementary Material**. Further inquiries can be directed to the corresponding author.

## Supplementary material

Supplementary Figure 1 Correlation analysis of CPZ with Carcinoma-Associated Fibroblast (CAF) infiltration in different cancers. **(A)** Heatmap of the fibroblast infiltration level of CPZ expression associated with pan-cancer with different algorithms. In gastric cancer (GC), cholangiocarcinoma (CHOL), and breast cancer (BC), CPZ gene expression was significantly positively correlated with CAF infiltration levels (P < 0.05). **(B)** Scatter plots showing the correlation between CPZ expression and fibroblast infiltration levels under different algorithms in GC conditions indicate that CPZ expression remains significantly positively correlated with fibroblast infiltration levels in gastric cancer (GC) (P < 0.01).
