# Supplementary figures and images for: Novel perceptions of the involvement of CPZ in gastric cancer prognosis and immunomodulation
Source: Front Oncol. 2025 Sep 10;15:1599542. doi: 10.3389/fonc.2025.1599542 (PMC12457134; doi:10.3389/fonc.2025.1599542)

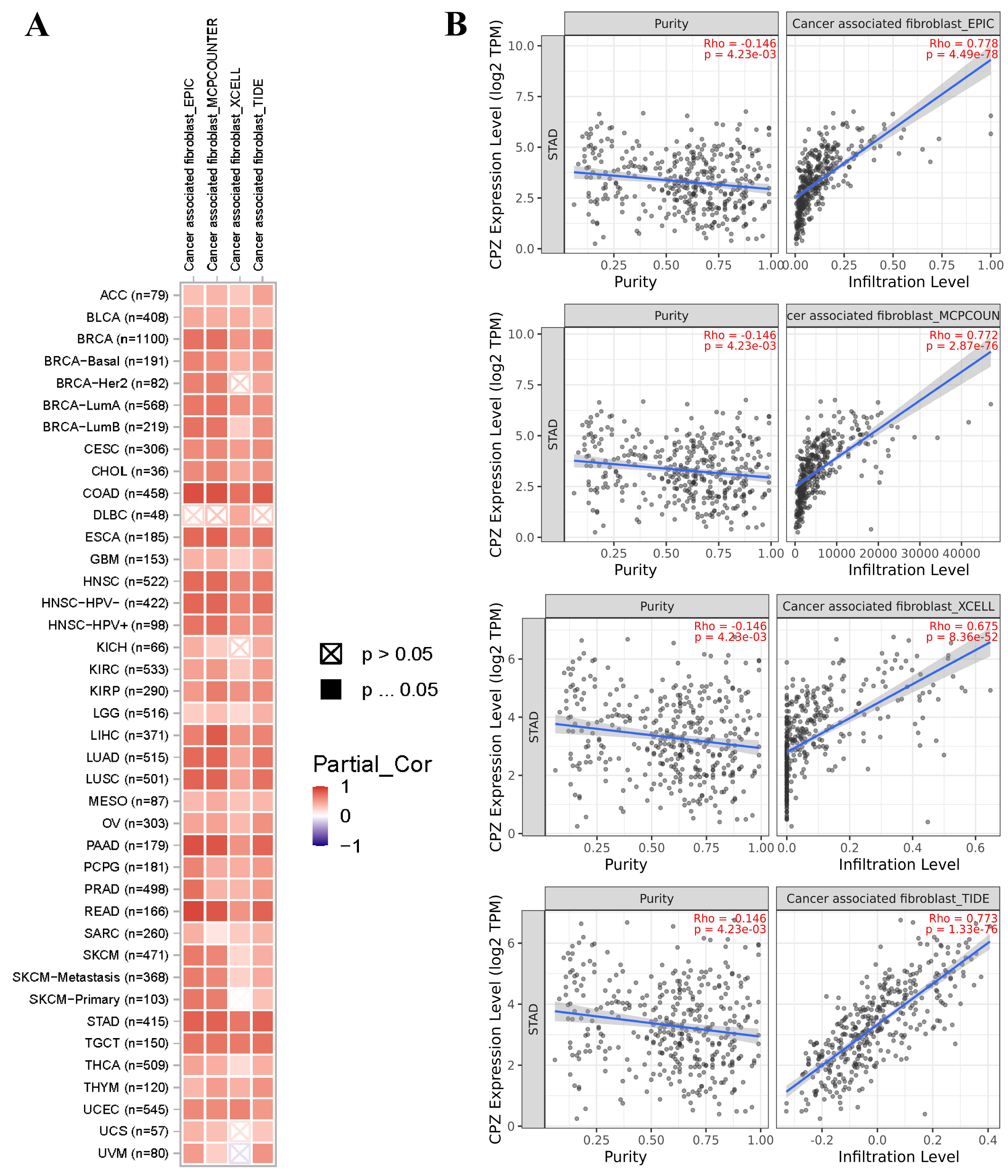

Supplement: Supplementary Figure 1 — Correlation analysis of CPZ with Carcinoma-Associated Fibroblast (CAF) infiltration in different cancers. (A) Heatmap of the fibroblast infiltration level of CPZ expression associated with pan-cancer with different algorithms. In gastric cancer (GC), cholangiocarcinoma (CHOL), and breast cancer (BC), CPZ gene expression was significantly positively correlated with CAF infiltration levels (P < 0.05). (B) Scatter plots showing the correlation between CPZ expression and fibroblast infiltration levels under different algorithms in GC conditions indicate that CPZ expression remains significantly positively correlated with fibroblast infiltration levels in gastric cancer (GC) (P < 0.01). [file Image1.tif]
